# Factors associated with delayed defibrillation in cardiopulmonary resuscitation: A prospective simulation study

**DOI:** 10.1371/journal.pone.0178794

**Published:** 2017-06-08

**Authors:** Christoph Castan, Alexander Münch, Moritz Mahling, Leopold Haffner, Jan Griewatz, Anne Hermann-Werner, Reimer Riessen, Jörg Reutershan, Nora Celebi

**Affiliations:** 1 Medical School, Faculty of Medicine, University of Tuebingen, Tuebingen, Germany; 2 Department of Anesthesiology and Intensive Care Medicine, University of Tuebingen, Tuebingen, Germany; 3 Department of Internal Medicine, Division of Endocrinology, Diabetology, Vascular Disease, Nephrology and Clinical Chemistry, University of Tuebingen, Tuebingen, Germany; 4 Competence Centre for University Teaching in Medicine, Faculty of Medicine, University of Tuebingen, Tuebingen, Germany; 5 DocLab, Faculty of Medicine, University of Tuebingen, Tuebingen, Germany; 6 Department of Internal Medicine, Psychosomatic Medicine and Psychotherapy, University of Tuebingen, Tuebingen, Germany; 7 Department of Internal Medicine, Medical Intensive Care Unit, University of Tuebingen, Tuebingen, Germany; 8 Department of Anesthesiology and Intensive Care Medicine, Bayreuth Hospital, Preuschwitzer Straße 101, Bayreuth, Germany; 9 PHV-Dialysezentrum Waiblingen, Beinsteiner Straße 8/3, Waiblingen, Germany; Azienda Ospedaliero Universitaria Careggi, ITALY

## Abstract

**Introduction:**

Early defibrillation is an important factor of survival in cardiac arrest. However, novice resuscitators often struggle with cardiac arrest patients. We investigated factors leading to delayed defibrillation performed by final-year medical students within a simulated bystander cardiac arrest situation.

**Methods:**

Final-year medical students received a refresher lecture and basic life support training before being confronted with a simulated cardiac arrest situation in a simulation ambulance. The scenario was analyzed for factors leading to delayed defibrillation. We compared the time intervals the participants needed for various measures with a benchmark set by experienced resuscitators. After training, the participants were interviewed regarding challenges and thoughts during the scenario.

**Results:**

The median time needed for defibrillation was 158 s (n = 49, interquartile range: 107–270 s), more than six-fold of the benchmark time. The major part of total defibrillation time (49%; median, n = 49) was between onset of ventricular fibrillation and beginning to prepare the defibrillator, more specifically the time between end of preparation of the defibrillator and actual delivery of the shock, with a mean proportion of 26% (n = 49, SD = 17%) of the overall time needed for defibrillation (maximum 67%). Self-reported reasons for this delay included uncertainty about the next step to take, as reported by 73% of the participants. A total of 35% were unsure about which algorithm to follow. Diagnosing the patient was subjectively difficult for 35% of the participants. Overall, 53% of the participants felt generally confused.

**Conclusions:**

Our study shows that novice resuscitators rarely achieve guideline-recommended defibrillation times. The most relative delays were observed when participants had to choose what to do next or which algorithm to follow, and thus i.e. performed extensive airway management before a life-saving defibrillation. Our data provides a first insight in the process of defibrillation delay and can be used to generate new hypotheses on how to provide a timely defibrillation.

## Introduction

Sudden cardiac arrest is the worldwide leading cause of death and is often based on ischemic heart diseases [1,2]. The latest European Resuscitation Council Guidelines for Resuscitation 2015 recommend instant bystander cardiopulmonary resuscitation and early electrical defibrillation [2]. Up to 76% of patients who have cardiac arrest initially present with ventricular fibrillation (VF), eventually deteriorating into asystole [2]. In contrast to an overall survival rate after (out-of-hospital) cardiac arrest of 8%, the survival rate after initial VF and early defibrillation is up to or higher than 20% [2,3]. Early defibrillation has been linked to an improvement of patient outcome when suffering from VF or ventricular tachycardia [4]. *Chan et al*. showed a loss of likelihood of survival and hospital discharge and an increased likelihood of neurological disabilities after delayed defibrillation [5].

Therefore, electrical therapy should be performed as soon as possible within a shockable cardiac rhythm. For an in-hospital cardiac arrest situation, defibrillation is recommended within 2 min after recognition [5,6]. Although there are clear and standardized procedural recommendations available for cardiac arrest situations, early defibrillation appears to pose a problem for novice resuscitators. *Hunt et al*. observed a prolonged pre-shock pause of 84 s [7]. They also showed that within a simulation study with third-year pediatric residents as participants, 7% did not defibrillate at all. Further studies have detected different factors for delayed defibrillation, including in-hospital cardiac arrest, hospital bed size, unmonitored hospital units, and non-cardiac hospital admission [5].

A reliable database concerning the effect of the physician’s experience in managing cardiac arrest situations is lacking. According to our observations during teaching basic and advanced life support lessons to final-year medical students, timely defibrillation appears to pose a challenge to most students. Therefore, we conducted a prospective simulation study aiming to identify factors leading to delayed defibrillation by novice resuscitators.

## Materials and methods

### Study design

We used a prospective study design to assess the time required by final-year medical students to perform successful defibrillation after onset of VF within a simulated cardiac arrest scenario. The study was conducted from January to March 2015 at the University of Tuebingen, Germany.

### Study participants and ethics

**Fig 1:**
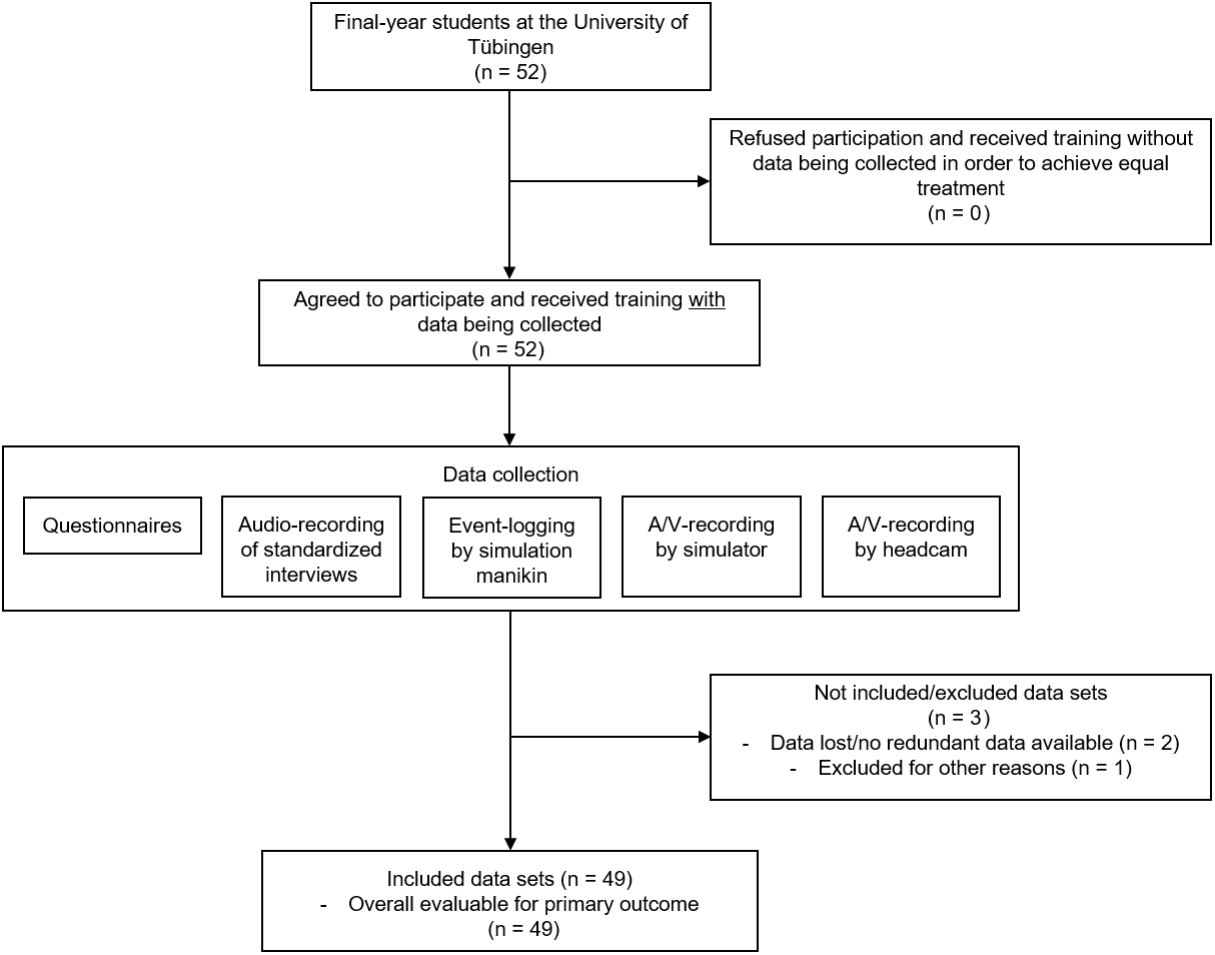
Acquisition, inclusion, and exclusion of probands. Arrows pointing right show exclusion from the study. Arrows pointing down indicate progress.

We asked a total of 52 final-year medical students (6^th^ year), attending curricular advanced life support training, to participate in this study (Fig 1). All of the participants gave their written consent. We excluded three datasets because of data loss and not performing one scenario. The study was approved by the ethical committee of the University of Tuebingen (Reference 687/2014A).

### Resuscitation training

The training day was divided into two parts as follows: refreshment of theoretical knowledge (35-min refresher lecture, see S1 File) and basic life support training (15 min) (based on recommendations of European Resuscitation Council Guidelines 2010 [8]), followed by an individual training session with subsequent feedback (S1 Fig). We briefed the participants about handling and operation of the defibrillator, including risks and safety instructions (10 min). We showed them how to establish electrocardiogram (ECG) leads, how to apply defibrillation patches to the mannequin, and how to safely deliver a shock.

Following the introductory seminar on use of the defibrillator (LifePak 15; PhysioControl Inc., Redmond, WA, USA), the students underwent familiarization with the equipment of the simulation ambulance. This familiarization included location and handling of airway devices, drugs, the mannequin (Resusci Anne Simulator, Laerdal Medical GmbH, Puchheim, Germany), and further provided equipment.

### Training environment (simulation ambulance)

We used a simulation ambulance for the training (S2 Fig). The simulation environment comprised a realistic interior with fully functional equipment and a full-scale mannequin.

### Simulated cardiac arrest scenario

Within the simulated cardiac arrest scenario of acute chest pain and dyspnea (S3 Fig), the participants assumed the role of an emergency physician on an ambulance with the support of a standardized assistant, i.e. a paramedic. The paramedics’ skills were restricted (i.e. defibrillation or airway management had to be performed by the participant).

During the simulated scenario, the paramedic supported the participant, but was not allowed to influence the scenario by providing medical advice or taking action autonomously. The assistants were instructed to provide standardized decline answers in case of being instructed to perform a restricted task. A comprehensive list of allowed and restricted tasks is shown in S1 Table.

### Qualitative interview, questionnaire and video review

After finishing the scenario, the participants completed a standardized questionnaire and standardized interview (S2 File). At this point, standardized data collection was complete and the participants received individual feedback for the resuscitation performance in the first training scenario (“debriefing”). This non-standardized feedback was not meant to be analyzed and therefore wasn’t recorded. The questionnaires and interviews were analyzed afterwards (see Results below). The videotapes were reviewed to detect common mistakes or errors leading to a delayed defibrillation.

### Outcomes

The primary outcome was the absolute period of time until a shock was delivered to the patient after onset of VF. We measured and analyzed this period of time using different tools simultaneously as follows. Two raters independently analyzed the recordings of the training session. For redundancy and accuracy, we also matched these timecodes with log files produced by the simulation manikin. Based on these timecodes, we calculated the absolute amount of time to defibrillation.

**Fig 2:**
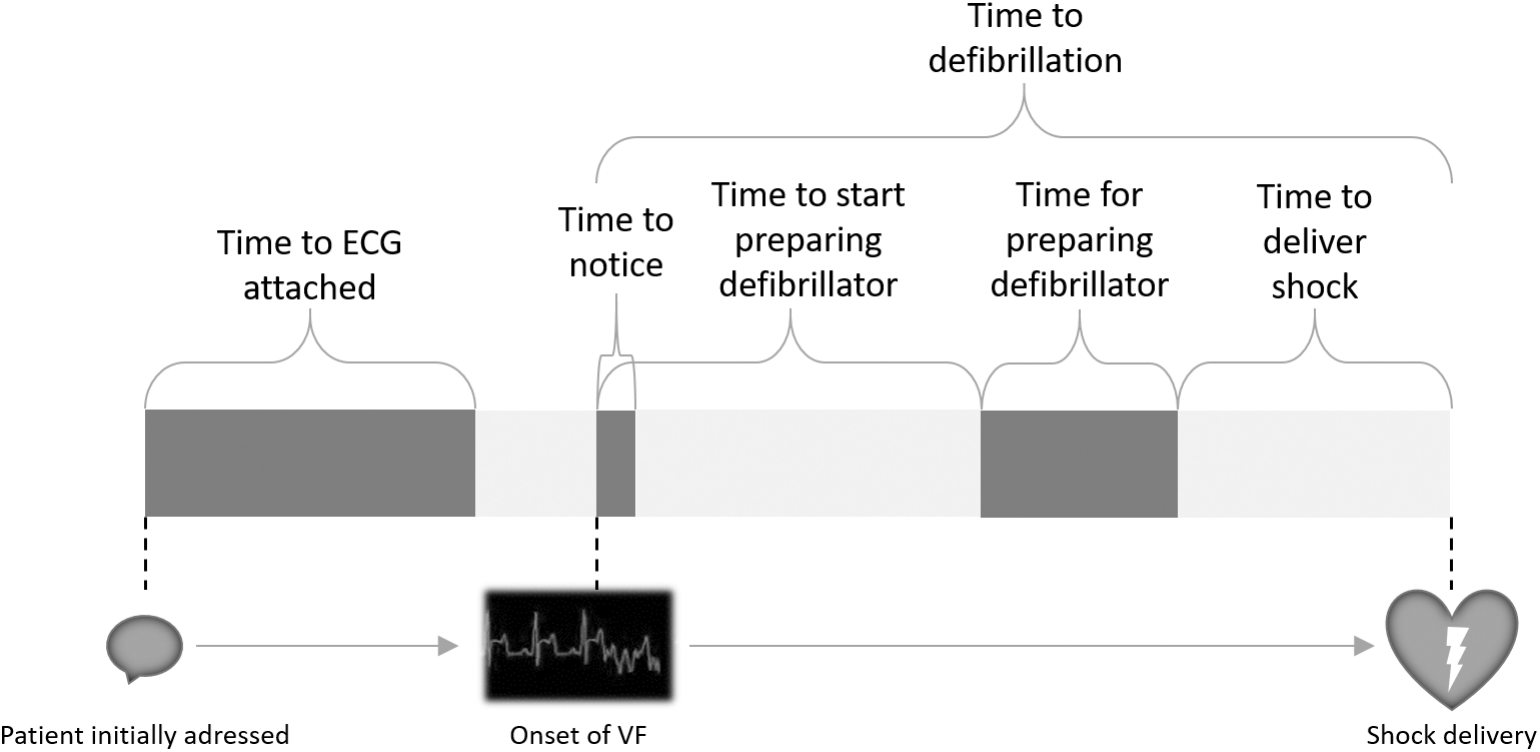
Sub-intervals of time to defibrillation. The scenario starts with initially addressing the patient (left) and ends with delivering the shock (right). Proportions of the diagram do not represent actual values.

The scenario sequence was further divided into the following parts (Fig 2): **1.** Amount of time passed until ECG leads were completely established (electrodes applied to patient’s skin and monitor activated); **2.** Time passed after visible change in ECG until the participant noticed the new acute problem; **3.** Time passed after onset of VF until the participant started to prepare the defibrillator; **4.** Time needed by the participant for completely preparing the defibrillator (turning it on and applying the defibrillation patches); **5.** Time passed after completion of preparation of the defibrillator until delivery of the shock.

Furthermore, we correlated the period until defibrillation with comments from the participants, and inquired about prior specialized knowledge (traineeship or job experience).

To set a benchmark for the minimal time needed to perform the defibrillation, two experienced resuscitators conducted a benchmark test under the standardized scenario setting. The time between first monitoring the alarm of VF and performing defibrillation was videotaped and analyzed.

### Statistical analysis

All results are presented descriptively. Normally distributed results are shown as mean with standard deviation (SD). Data that did not follow a normal distribution are shown as median with 25–75% quartiles (Q_25_–Q_75_).

## Results

**Fig 3:**
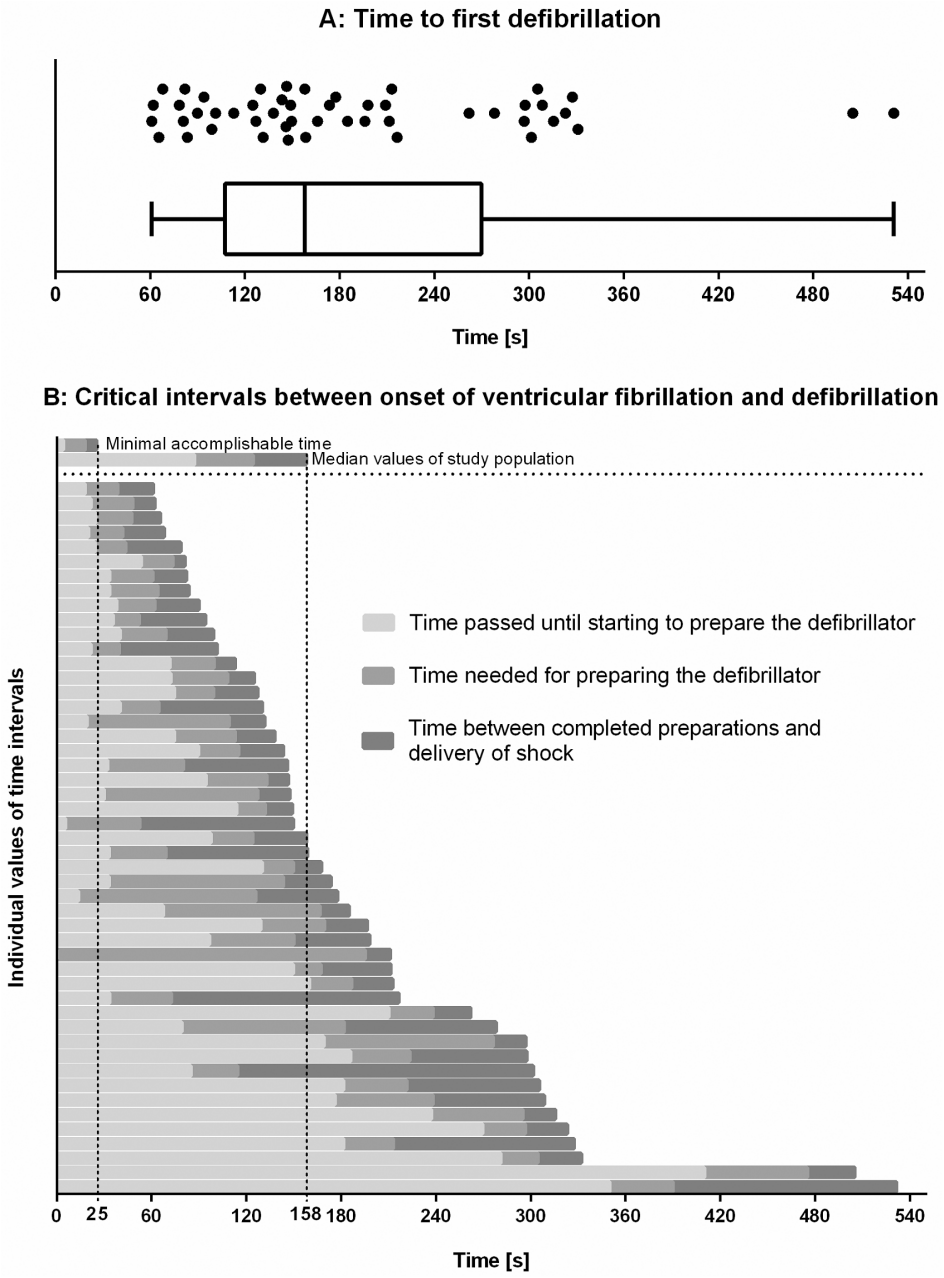
**A:** Time needed for the first defibrillation after onset of ventricular fibrillation (n = 49). **B:** Stacked-bar summary of critical intervals between the onset of ventricular fibrillation and the first defibrillation. Minimal accomplishable time (25 s, determined in-house by experienced personnel) and median values of the study population are shown in the top rows for comparison.

Table 1 shows the baseline characteristics of the participants (n = 49). Every participant attended at least one curricular advanced life support course during the 3^rd^ year of studies, the median interval until the last training was 24 months. The time between onset of cardiac arrest and delivery of the electrical defibrillation was defined as the time to defibrillation. The median time to defibrillation in this cohort was 158 s (Q_25–_Q_75_: 107–270 s) (Fig 3A) with a minimal value of 25 s in the benchmark test. The distribution presented as left-skewed. The value of 158 s represented 6.3-fold of the benchmark time.

**Table 1:** Characteristics of the participants.

Total, n	49
Age in years, median (25% and 75% quartiles)	26 (26–29.5)
Female sex, n (%)	28 (57%)
Month since last resuscitation training, median (25% and 75% quartiles)	24 (12–30)
Paramedical qualification, n (%)	4 (8%)
Other medical qualification, n (%)	5 (10%)

We divided the time to defibrillation into smaller fractions. A total of 72 s (median, Q_25–_Q_75_: 33–155 s) elapsed between the onset of VF and beginning to prepare the defibrillator (72 s = 18-fold of the benchmark time [4 s]). Preparing the defibrillator took 31 s (Q_25–_Q_75_: 24–51 s), which was 2,2-fold of the benchmark time (14 s). The time between finishing preparation for a defibrillation and actually releasing the electrical shock was a median of 28 s (Q_25–_Q_75_: 21–64 s), which was 4-fold of the benchmark time (7 s; Fig 3A and 3B).

All videotapes were reviewed to detect common problems and mistakes that lead to delayed defibrillation (Table 2). The following major problems were detected: Actions distracting from preparing and performing defibrillation (i.e., extensive airway management) were performed before the first defibrillation in 16 scenarios (33% of the participants). Another observation was that although the defibrillator was fully prepared, the delivery of the shock was substantially delayed (n = 16, 33%).

**Table 2:** Observed factors in video analysis potentially leading to delayed defibrillation and self-reported problems mentioned by the participants.

**Observed factors in video analysis potentially leading to delayed defibrillation**		
**Problem category**	**Detailed problem**	**Observed (no. of participants)**
Prioritization/order of measures	Monitoring: missing, incomplete, or delayed (blood pressure, oxygen saturation, ECG)	51% (25)
	Noticeable delay between finishing preparation of the defibrillator and actually delivering the shock	33% (16)
	Extensive airway management prior to defibrillation	33% (16)
	No CPR provided	14% (7)
Crisis/crew resource management	CPR provided by the participant/not delegated to assistant, thus blocking participant from setting up the defibrillator	24% (12)
**Self-reported problems mentioned by the participants**		
**Problem category**	**Detailed problem**	**Reported (no. of participants)**
Prioritization	The next step was unclear	73% (36)
	Choice of applicable algorithm unclear	35% (17)
Coordination	General/unspecific confusion	53% (26)
Diagnosis and medication	Diagnosis was difficult	35% (17)
	Insecurity with medication	22% (11)
Material and Personnel	Operation/handling (of devices) was difficult	14% (7)
	Insufficient number of helpers	10% (5)

During the standardized interview after the scenario, the students were asked about problems or difficulties within the simulation scenario. The manifest content was coded into 24 categories. All problems related to simulation artifacts, such as unrealistic behavior or malfunction of the mannequin, were excluded. Items mentioned by more than 10% of the participants were clustered into the categories of “prioritization”, “coordination”, “diagnosis and medication” and “other”, as shown in Table 2. In particular, difficulties in the prioritization category, such as the next step was unclear (73%) and not knowing which algorithm should be applied (35%), were verbalized by the students. Another problem of general unspecific confusion was mentioned in 53% of all interviews.

## Discussion

In this prospective study, we investigated different factors affecting the time to defibrillation in a simulated cardiac arrest scenario. For an in-hospital cardiac arrest, the recommended time to defibrillation should be under 2 min [5] respectively 3 min [4]. We found a median time to defibrillation of 158 s (2.6 min), with a large variance in time frame. Similar results were reported by Sullivan et al., who found that nurses eventually defibrillated after a delay of 109–157 s [9].

In contrast, our benchmark yielded a minimal achievable time to defibrillation of 25 s. Compared with this benchmark, the median defibrillation delay of our study group was approximately 2 min longer. This delay could potentially negatively affect the patient’s outcome [2,4]. This is supported by clinical experience showing that an immediate defibrillation yields a high defibrillation success rate [10].

By dividing the time to defibrillation in clinically important sub-intervals, we analyzed the causes for the delay in defibrillation. The major portion of time for defibrillation was the time between onset of VF and starting to prepare the defibrillator (median, 72 s). The majority of participants rapidly noticed the new situation (2.5 s, IQR 2.0–6.5 s), which is likely due to the immediate alarm from the vital sign monitor. Six (12%) participants had no ECG established until onset of VF, and monitoring of patients was incomplete in 25 scenarios at this time. Our analysis suggests that establishing ECG leads too late (after onset of VF) was associated with a delayed start of defibrillation setup (median of 54 s when established before onset of VF vs. 115 s when established after onset of VF).

In our scenario, the next step was to prepare the defibrillator, which took a median of 31 s. In our benchmark, this step accounted for 14 s. This clearly indicated that our participants lacked experience in handling our defibrillator, although only 14% reported difficulties with handling of the device. Nevertheless, this situation might represent a daily issue of emergency personnel who do not defibrillate routinely. Possible approaches to reduce this time further include using commonly used devices, as well as intuitively and easy to use devices, such as automated external defibrillators in the training sessions.

After the defibrillator was prepared, we expected the participants to release the electrical shock shortly after with prior warning. In contrast to our expectations, this took a median of 28 s, which is four times longer compared with the benchmark value. An even longer delay between availability of the defibrillator and administration of an electrical shock of 45 s has also been observed by Marsch et al. [11].

While preparing the defibrillator took only 2,2-times longer compared to the benchmark (which could easily be affected by regular device handling), we were surprised that the longest relative delays were detected for *beginning to prepare the defibrillator after onset of VF* (18-fold, although VF was noticed almost instantly) and for *actually releasing the electrical shock* (4-fold). Using our subsequent video analysis and questionnaires, we could detect possible reasons for these delays. Many participants reported that “the next step” or the “choice of applicable algorithm” were unclear. One reason for this might have been uncertainty whether to follow the algorithm for a shockable vs. the algorithm for a non-shockable resuscitation, as both were discussed in the theory lesson prior to the simulation. We also identified technical reasons, as monitoring was missing, incomplete, or delayed in half of the scenarios. Furthermore, we observed extensive airway management prior to defibrillation in 16 scenarios (33%, Table 2). In line with this finding, the majority of participants reported issues with prioritization (i.e., the next step was unclear).

This “unorganized” approach may be explainable with the first stage of the Dreyfus model of skill acquisition [12]. This model postulates five different stages of development from novice to mastery. The first stage (novice) of this model is defined as “rigid adherence to taught rules or plans” with “little situational perception” and “no discretionary judgement” [12]. Besides stage one attributes, the participants also showed characteristics of stage two (advanced beginner), defined as having “situational perception” and “treating all aspects of work separately with equal importance” [12]. This could explain why we observed little to no fundamentally wrong treatment for an individual problem. The students might even have reached the competence stage (stage three), where too much relevant information and possible procedures are available and become overwhelming [12].

In summary, all participants should have been able to perform all necessary measures in a timely manner, but often failed to do so. In conclusion, participants did not act “straight forward” and regularly showed signs of uncertainty about what to do next. This is reflected in incomplete monitoring (which should be established first) and extensive airway management before life-saving defibrillation, and matches the self-reported “The next step was unclear” and “General confusion”. Our evidence is limited by simulation artifacts because most students had neither worked in a real ambulance nor worked with the provided equipment before. However, the simulation environment enabled us to perform a detailed analysis of our participants’ behavior. We conducted a theoretical refresher lecture prior to our simulation to achieve a comparable knowledge level, this might however have conditioned the performance of our participants. However, this probably was necessary as a median of 24 months have elapsed since the last resuscitation training of our participants and our study, and literature documents a rapid decay of knowledge [13]. Regardless of the brief introduction to the environment and devices, the reasons stated above could have led to an excessive additional cognitive demand. This demand could have caused 53% of the participants to mention general confusion.

## Conclusion

In this prospective, qualitative study, we identified factors that affect the time to defibrillation in a simulated cardiac arrest scenario. Overall, final-year medical students needed 158 s after onset of VF to deliver a potentially life-saving shock compared with 25 s in a benchmark test. We identified uncertainty about which algorithm to follow and a lack of prioritization as the main reasons for this finding, and almost one-quarter of the time to defibrillation was lost after the defibrillator was already in place and fully set up. A possible approach to the solution of the problems mentioned above could be to focus on teaching core resuscitation elements and algorithm choice first. Supporting factors, such as extended airway management and medication, could be added in a follow-up training.

## Supporting information

S1 Fig Training day sequence for three participants. The participants received a refresher lecture at the beginning and basic life support training. They then worked sequentially through the simulated cardiac arrest scenario and received immediate feedback.
(TIF)

S2 Fig Perspective of the back of a simulation ambulance. The numbers in the figure indicate the following: (1) simulation mannequin (Resusci Anne Simulator; Laerdal Medical GmbH, Puchheim, Germany), (2) defibrillator (LifePak 15; PhysioControl Inc., Redmond, WA, USA), (3) ventilation bag with filter and mask, (4) supplied set for intravenous drug administration, (5) simulated intensive care monitor showing the patient’s vitals (Patient Monitor; Laerdal Medical GmbH), and (6) suction unit (AccuVac Rescue; Weinmann, Germany).
(TIF)

S3 Fig Sequence of the simulated cardiac arrest scenario. The participants triggered the 3.5-min countdown to cardiac arrest while initially addressing the patient. After the first defibrillation, the patient achieved return of spontaneous circulation.
(TIF)

S1 Table List of allowed and restricted tasks for the standardized resuscitation assistants. The defibrillator charge was restricted to 5 joules for safety reasons.
(DOCX)

S1 File Lecture slides. (PDF)

S2 File Supporting information. Questionnaires.
(DOCX)

S3 File Supporting information. Original Data Set.
(XLSX)
