# Supplementary figures and images for: Factors associated with delayed defibrillation in cardiopulmonary resuscitation: A prospective simulation study
Source: PLoS One. 2017 Jun 8;12(6):e0178794. doi: 10.1371/journal.pone.0178794 (PMC5464587; doi:10.1371/journal.pone.0178794)

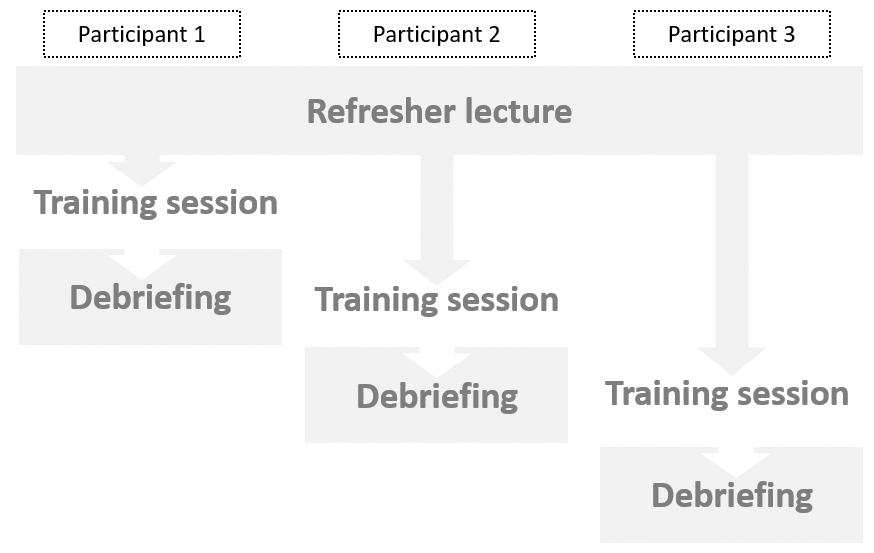

Supplement: S1 Fig — The participants received a refresher lecture at the beginning and basic life support training. They then worked sequentially through the simulated cardiac arrest scenario and received immediate feedback. (TIF) [file pone.0178794.s001.tif]

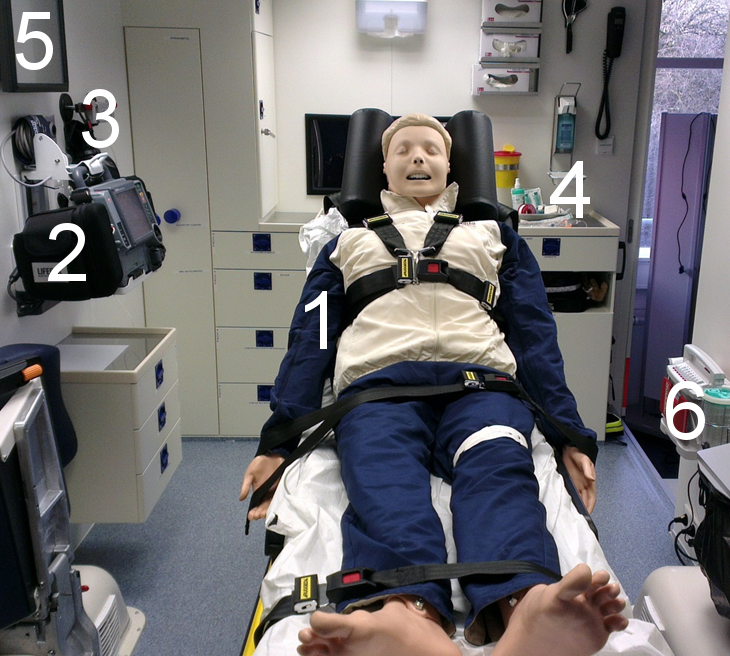

Supplement: S2 Fig — The numbers in the figure indicate the following: (1) simulation mannequin (Resusci Anne Simulator; Laerdal Medical GmbH, Puchheim, Germany), (2) defibrillator (LifePak 15; PhysioControl Inc., Redmond, WA, USA), (3) ventilation bag with filter and mask, (4) supplied set for intravenous drug administration, (5) simulated intensive care monitor showing the patient’s vitals (Patient Monitor; Laerdal Medical GmbH), and (6) suction unit (AccuVac Rescue; Weinmann, Germany). (TIF) [file pone.0178794.s002.tif]

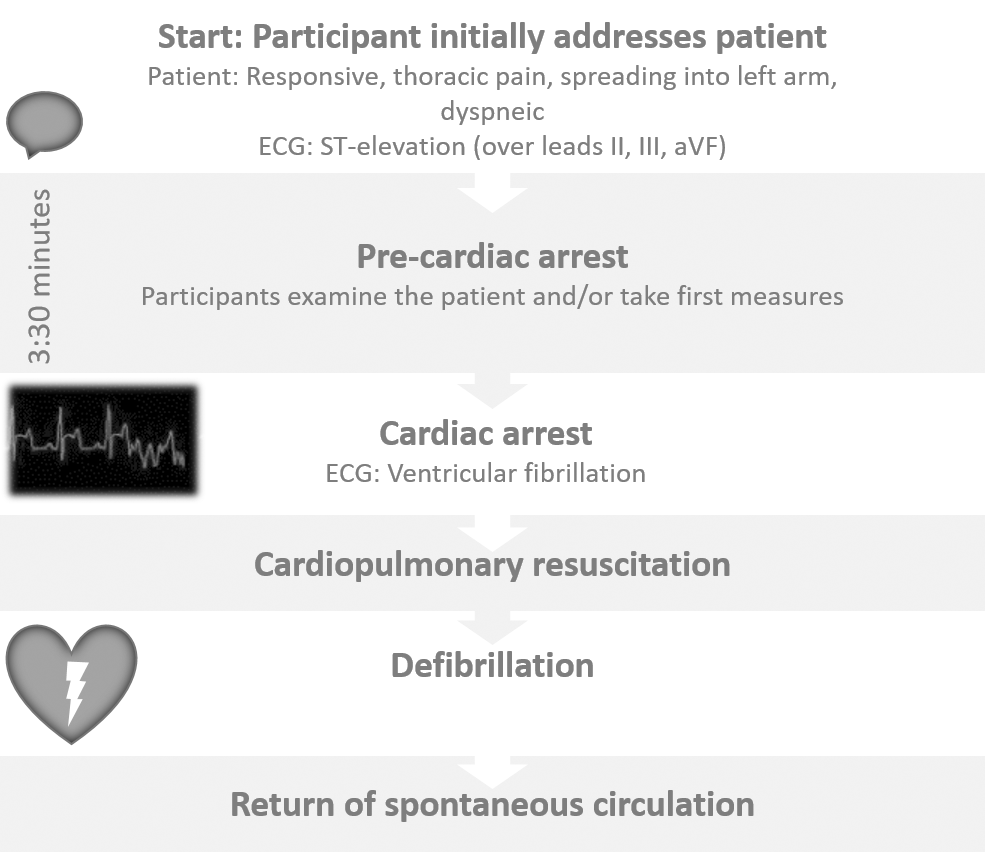

Supplement: S3 Fig — The participants triggered the 3.5-min countdown to cardiac arrest while initially addressing the patient. After the first defibrillation, the patient achieved return of spontaneous circulation. (TIF) [file pone.0178794.s003.tif]
