# Supplementary material for: Factors associated with delayed defibrillation in cardiopulmonary resuscitation: A prospective simulation study
Source: PLoS One. 2017 Jun 8;12(6):e0178794. doi: 10.1371/journal.pone.0178794 (PMC5464587; doi:10.1371/journal.pone.0178794)
Supplement: S1 Table — The defibrillator charge was restricted to 5 joules for safety reasons. (DOCX) [file pone.0178794.s004.docx]

| **Situation/instruction** | **Assistant’s answer (analogous)** |
| --- | --- |
| Assistant is instructed to apply the defibrillation patches/to set up the defibrillator | “I am not trained to use this device” |
| Assistant is instructed to analyze the ECG | “ECG analysis was not part of my training so far” |
| Assistant is instructed to secure the airway/intubate | “I have never intubated before” or “I have not been trained to perform this measure” |
| Assistant is instructed to perform defibrillation | “I have not been instructed how to do this. Perhaps we should switch our tasks.” |
| Defibrillator is fully charged, but no warning is given/defibrillator is charged to more than 5 joules | Loud announcement of “STOP” 🡪 Safety break of scenario. |
| Participant asks for drug dosage, order of measures, or general medical advice | “I don’t know“ or “I never witnessed a real resuscitation before” |
