# Supplementary material for: Factors associated with delayed defibrillation in cardiopulmonary resuscitation: A prospective simulation study
Source: PLoS One. 2017 Jun 8;12(6):e0178794. doi: 10.1371/journal.pone.0178794 (PMC5464587; doi:10.1371/journal.pone.0178794)
Supplement: S1 File — (PDF) [file pone.0178794.s005.pdf]

EBERHARD KARLS UNIVERSITÄT TÜBINGEN

DocLab Simulationszentrum
Medizinische Fakultät Tübingen

UNIVERSITÄTS KLINIKUM TÜBINGEN
Universitätsklinik für Anästhesie und Intensivmedizin

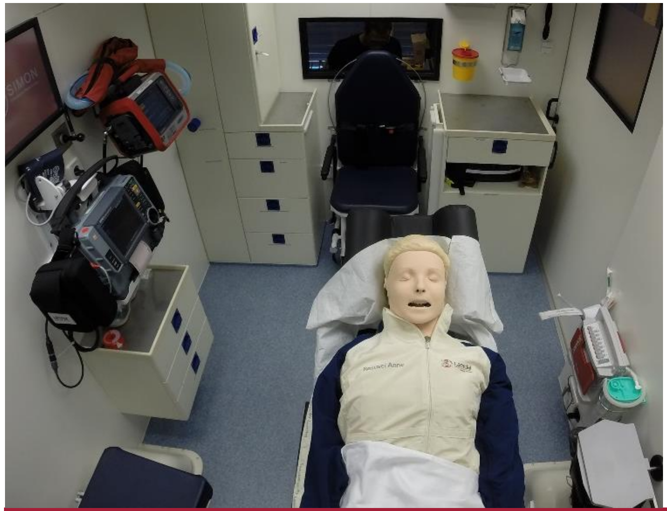

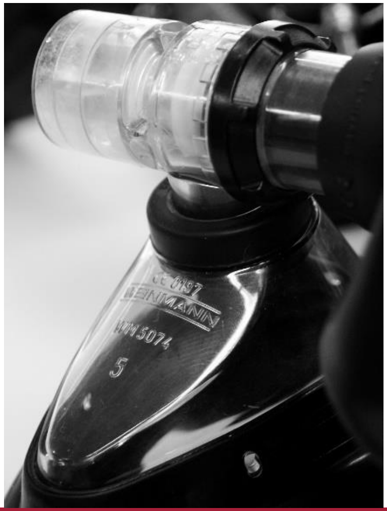

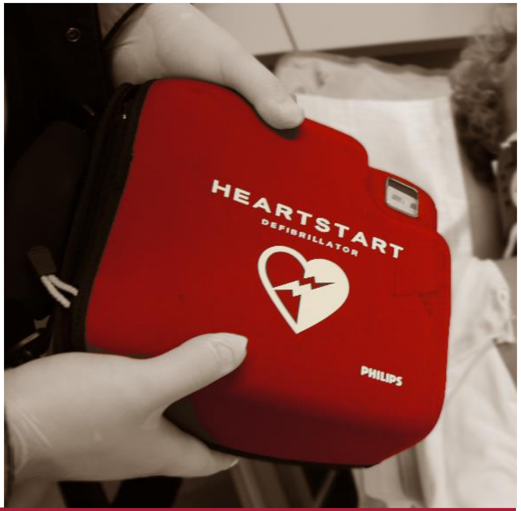

Resuscitation-Training

(Translated version of original presentation)

Schedule

Preparation
- Theoretical and practical skill training

Introduction to Simulator

Training 1
- Individual training
- Debriefing

Break

Training 2
- Team training
- Debriefing

© 10.11.2016 Universität Tübingen

Advanced Life Support

BLS	Material	Algorithm / Teamwork

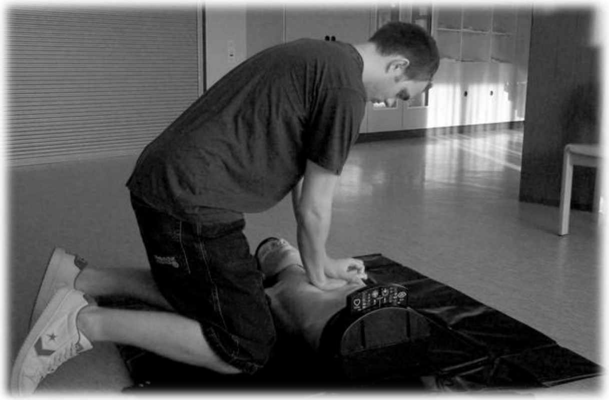

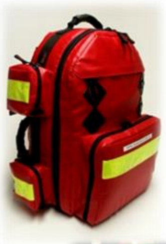

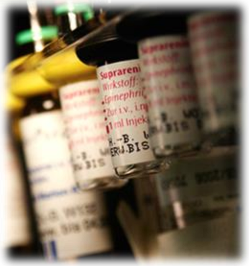

© 10.11.2016 Universität Tübingen

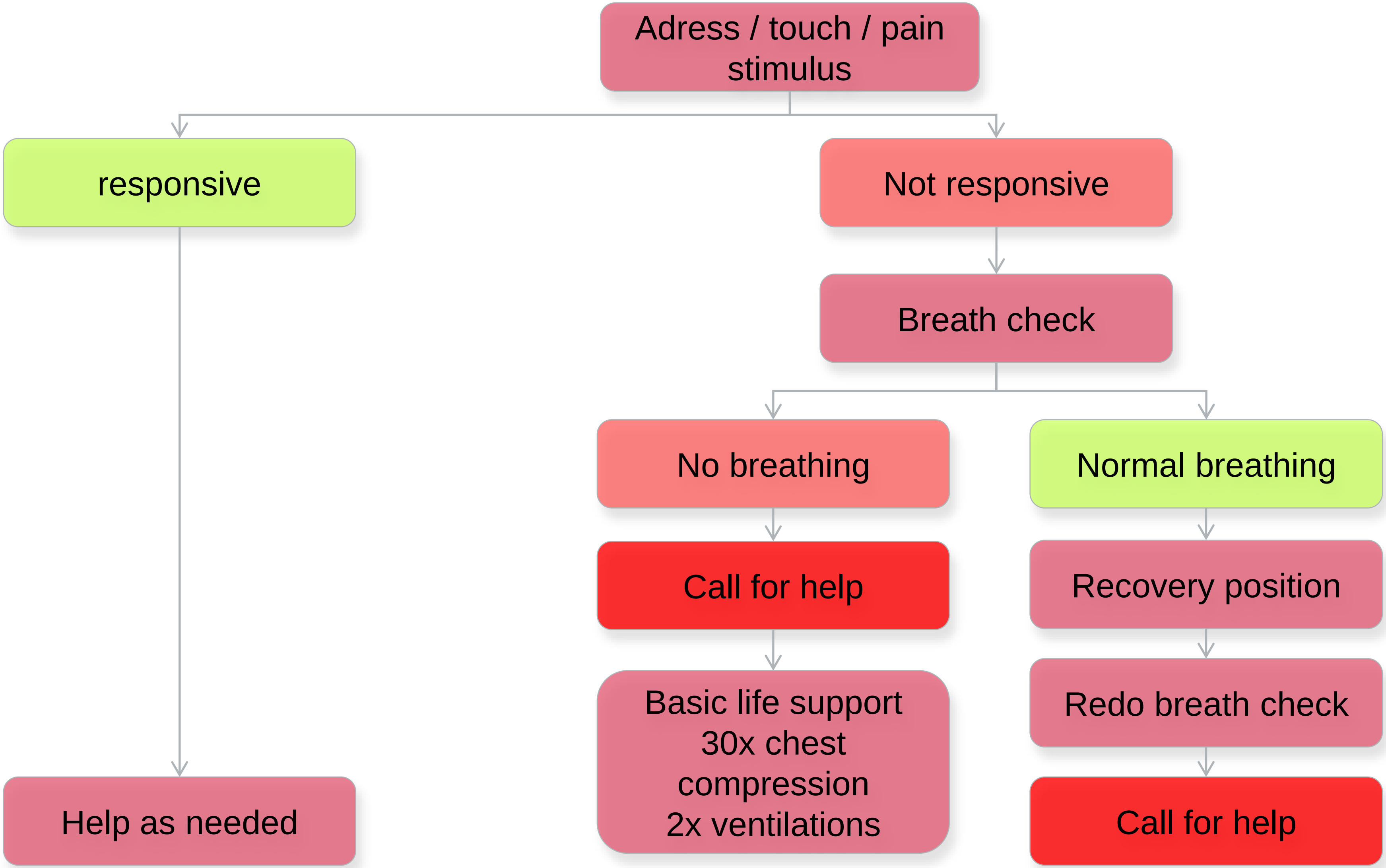

© 10.11.2016 Universität Tübingen

Basic Life Support
BLS

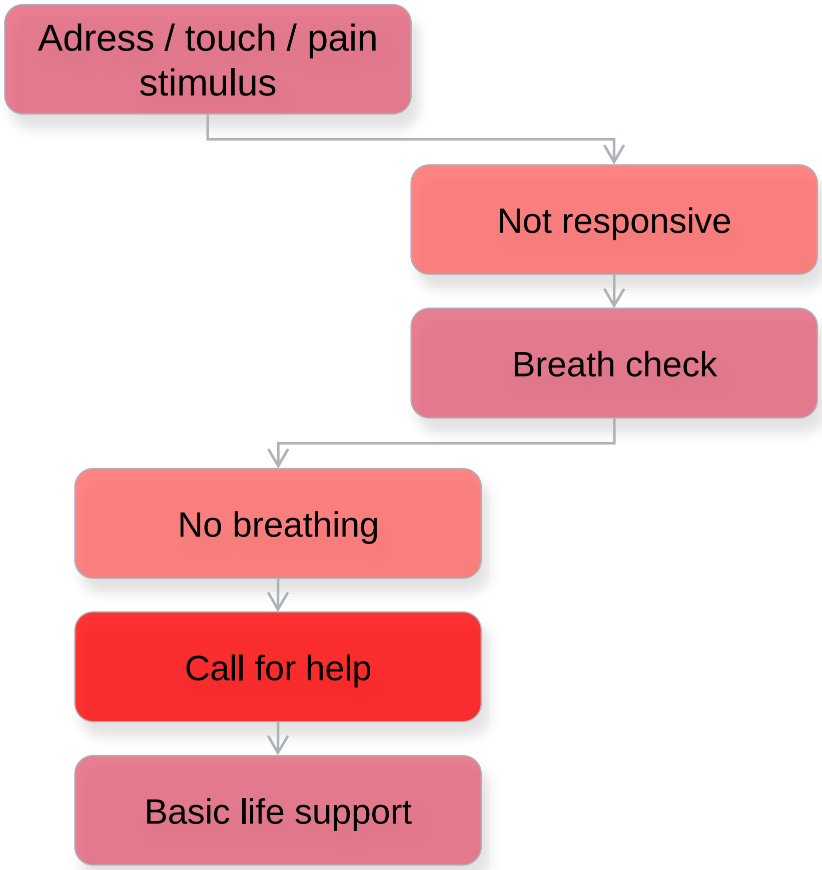

EBERHARD KARLS UNIVERSITÄT TÜBINGEN

UNIVERSITÄTS KLINIKUM TÜBINGEN

Cardio-pulmonary Resuscitation (CPR)

- Rhythm: 30 : 2
  - 30 chest compressions
  - 2 ventilations
- frequency: 100-120/ min
- depth: 5 – 6 cm
- Pressure point: lower half of sternum, medial

→ Minimal flow for oxygen supply

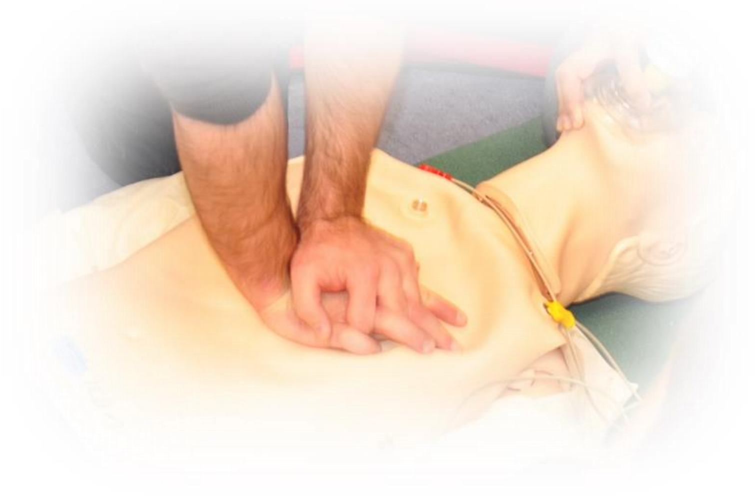

Ventilations

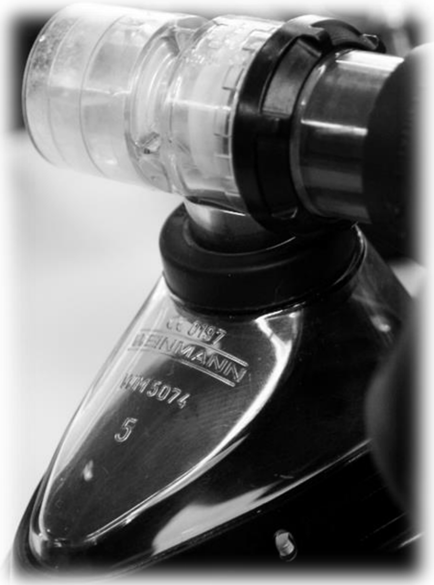

- **General**:
  - Ventilation with pure O_2 (Target-SpO2 94-98%)
  - Have suction unit at hand
  - Prepare alternative devices for airway protection
- **Bag-mask-ventilation**
  - 1-2 seconds per ventilation
  - Minimize hands-off-time
  - Consider Guedel-airway-tube
- **Endotracheal Intubation**
  - Goldstandard, but recommended to be performed by experienced personnel
  - Continuous chest conpressions with asynchronous ventilations (10 ventilations / min)
- **Supraglottic airway devices**
  - Laryngeal tube
  - Laryngeal mask

ECG

- **Shockable**
  - Ventricular fibrillation (VF)

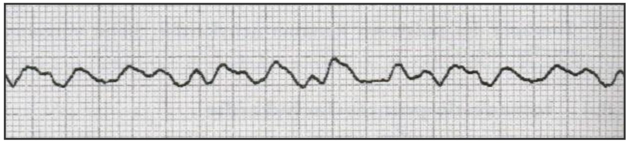

  - **Pulseless** ventricular tachycardia (pVT)

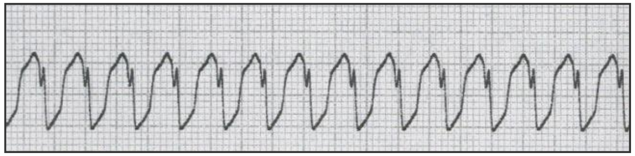

- **NOT shockable:**
  - Pulseless electric activity (PEA)

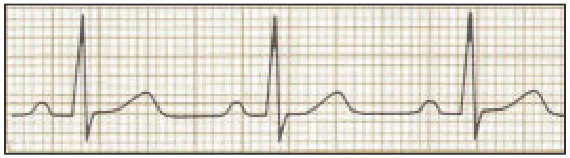

  - Asystole

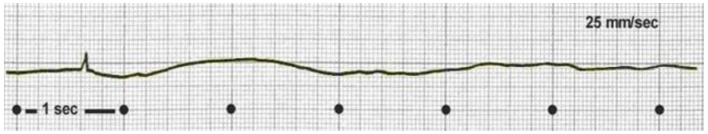

Defibrillation

- **Adult**
  - Dual-phasic
    - Initial: 150 – 200 J
    - All following shocks: maximum energy
  - Mono-phasic
    - Every shock: 360 J or maximum energy
    - Outdated technology, but devices still exist

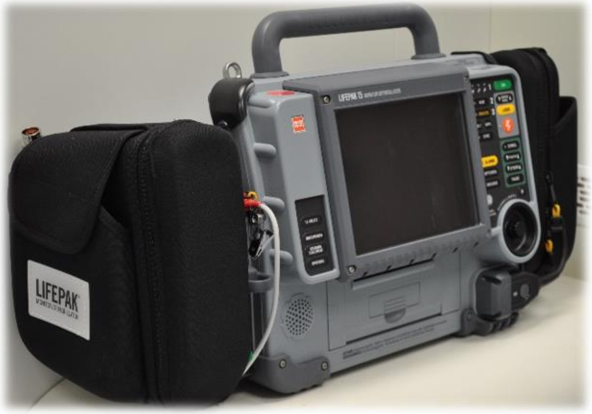

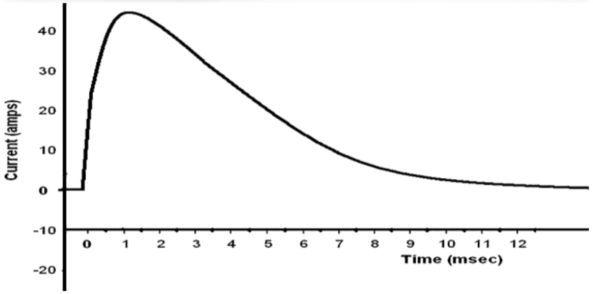

Abb. 1: Monophasische Defibrillation

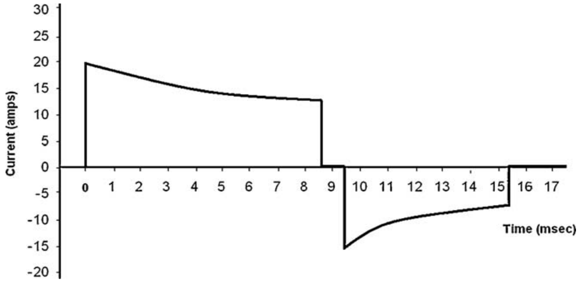

Abb. 2: Biphasische Defibrillation

Drugs

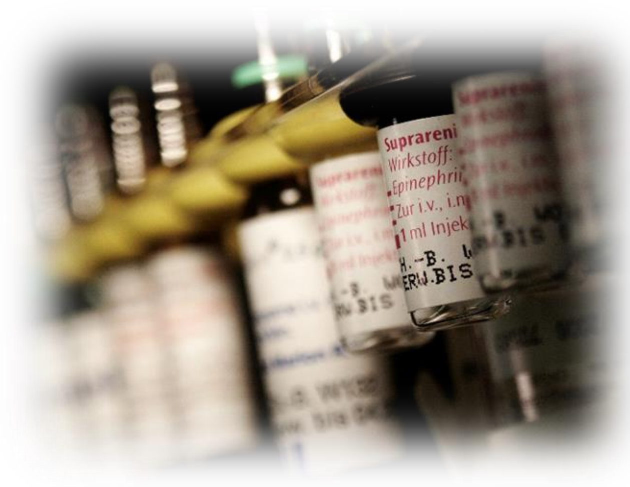

- Adrenalin (Suprarenin)
  - 1mg 1:10 diluted (1ml + 9ml NaCl)
  - Repeat every 3-5 min when appropriate
- Amiodaron (Cordarex)
  - 300mg (2 ampules) diluted with glucose 5% to 20 ml
  - Administer after 3rd uneffective shock
  - Repeat 150mg when appropriate

Advanced Life Support Algorithmus

Unconsciousness

Check breathing and circulation

Circulatory arrest
CPR 30:2 until defibrillator is ready

Check heart rhythm

Defibrillation
VT / VF

1 Defibrillation

Immediately continue CPR 30:2 for 2 min.

During CPR:
- i.v.-access/i.o.-access
- Airwaymanagement & O_2
- Continuous chest compressions after airway is secured
- Adrenalin every 3-5 min
- Correct reversible causes (HITS or 4 H‘s/4 T‘s)
- Consider Amiodaron with pVT and VF

No Defibrillation
Asystole or PEA

Immediately continue CPR 30:2 for 2 min.

Source: Resuscitation Guidelines ERC 2010

Questions
