# Supplementary material for: Factors associated with delayed defibrillation in cardiopulmonary resuscitation: A prospective simulation study
Source: PLoS One. 2017 Jun 8;12(6):e0178794. doi: 10.1371/journal.pone.0178794 (PMC5464587; doi:10.1371/journal.pone.0178794)
Supplement: S2 File — Questionnaires. (DOCX) [file pone.0178794.s006.docx]

1. Please fill in your age ____(years), your actual semester (FS) ____
   numbers of semester at an university:____
2. Mark your sex: □ female □ male
3. Have you participated in the obligatory emergency medicine course (QB8)?

- Yes, at the University of Tuebingen. FS: ___
- Yes, at another university. FS: ___
- No, I did not participate. Reason: ______________________
- No, I participated at an alternative course: _________________________ FS: ___

1. Do you have a professional medical education?

- Yes, paramedic (Rettungsassistent) □ Yes, paramedic (Rettungssanitäter)
- Yes, the following: ______________________________
- No

1. Have you ever taken part in an resuscitation? If yes, when?

- Yes, ____ month ago □ No

1. Have you ever worked in an ambulance before?
   - Yes □ No
2. Do you participate in resuscitation trainings periodically?

- Yes, periodical trainings, approx. _____times / year
- No

1. When was your last resuscitation training?
   Approx. ___ month ago
2. How would you estimate your resuscitation skills before the training?
   (Mark an „X“ on the line)

|  |
| --- |
|  |

Poor Good

1. Do you think a resuscitation training might be useful at the beginning of the practical year?
   - Yes □ No
2. My expectations of this resuscitation training are ?

- Release of my duties
- Refreshment of resuscitation knowledge and skills
- No benefit
- No expectations

1. How would you estimate your resuscitation skills after the first scenario?

(Mark an „X“ on the line)

|  |
| --- |
|  |

Poor Good

1. Please evaluate the following statements:

|  | agree full | agree partially | no agree partially | no agree fully | Not applicable |
| --- | --- | --- | --- | --- | --- |
| During the scenario I have always had the control over the events | □ | □ | □ | □ | □ |
| My assigned assistant executed all of my orders as I expected | □ | □ | □ | □ | □ |
| The head camera distracted me | □ | □ | □ | □ | □ |
|  |  |  |  |  |  |
| Within the following measures I felt unsecure: | Total unsecure | Partially unsecure | Partially secure | Total secure | Not performed |
| Drug administration and dosage | □ | □ | □ | □ | □ |
| ECG interpretation | □ | □ | □ | □ | □ |
| Ventilation | □ | □ | □ | □ | □ |
| Defibrillation | □ | □ | □ | □ | □ |
| Chest compressions | □ | □ | □ | □ | □ |
| Work environment in the ambulance | □ | □ | □ | □ | □ |
| Priorization / order of actions | □ | □ | □ | □ | □ |

| □ Did not diagnosed rhythm | □ Torsade-de-pointes-tachykardia | □ Sinusrhythm | □ Ventricular tachycardia |
| --- | --- | --- | --- |
| □ Ventricular fibrillation | □ ST elevation infarction | □ pulseless electrical activity | □ Asystole |

1. What was the patients ECG rhythm after loss of consciousness?
2. Please estimate the time between occurrence of the cardiac arrest to your first defibrillation

approx. _____ min _____ sec

1. What were your priorities for the following actions after occurrence of unconsciousness of the patient?

- Ventilations
- Defibrillation
- Chest compressions
- i.v.-Access
- Drug administration
- Extensive airway management
- ECG analysis

| Fill in 1 item | 1. priority | 2. priority |
| --- | --- | --- |
| Person 1 |  |  |
| Person 2 |  |  |
